# Different strategies of altering the cell wall to study the impact that these introduced changes have on plant resistance to pathogens

**DOI:** 10.3389/fpls.2025.1680357

**Published:** 2025-10-13

**Authors:** Megan F. DeTemple, Olga A. Zabotina

**Affiliations:** Roy J. Carver Department of Biochemistry, Biophysics and Molecular Biology, Iowa State University, Ames, IA, United States

**Keywords:** cell wall, plant defense, cell wall-degrading enzymes, pathogenesis, plant immune signaling

## Abstract

The cell wall (CW) plays many vital roles in plant fitness, ranging from plant development to defense against pathogens. There are many strategies used to make modifications to the CW and observe the consequences it has on plant development, physiology, and resistance to biotic and abiotic stresses. The most common methods used to change the CW include altering CW synthesizing gene expression by producing gene knockouts or overexpression lines, treating with CW enzyme inhibitors, applying exogenous hydrolases, and expressing CW degrading enzymes (CWDEs) in the plant. Alterations in the CW can change plant responses to pathogens through a variety of mechanisms including inducing plant immune pathways, eliciting a variety of PTI responses or by making the CW more resistant to pathogen infection. This review will highlight the insights gained about CW-mediated resistance and the function of each type of polysaccharide in pathogenesis grouped by each CW-modifying technique. Finally, the different advantages and disadvantages of these approaches will be discussed.

## 1 Introduction

Plants interact with the outside world through a cell wall (CW) composed of a variety of polysaccharides, including cellulose, pectin, and hemicellulose (Lampugnani et al., 2018; Delmer et al., 2024). Cellulose microfibrils are interwoven together and crosslinked with pectin and hemicellulose. The CW provides shape and structure to the cell. CWs need to be both flexible during cell division and expansion, and rigid after cell differentiation (Cosgrove, 2005; Delmer et al., 2024). The CW is a dynamic structure built, modified, and broken down as the plant grows and develops (Cosgrove, 2005; Delmer et al., 2024). The CW also functions as a physical barrier from outside biotic and abiotic dangers (Sasidharan et al., 2011). However, the CW does not just function as a passive barrier, but it also plays an essential role in pathogen perception and response (Bellincampi et al., 2014; Swaminathan et al., 2022). For example, plants secrete CWDE inhibitors like polygalacturonase-inhibiting proteins (PGIPs), xylanase inhibitor proteins (XIPs), and the CW is reinforced with callose and lignin (Bellincampi et al., 2014).

**Figure 1:**
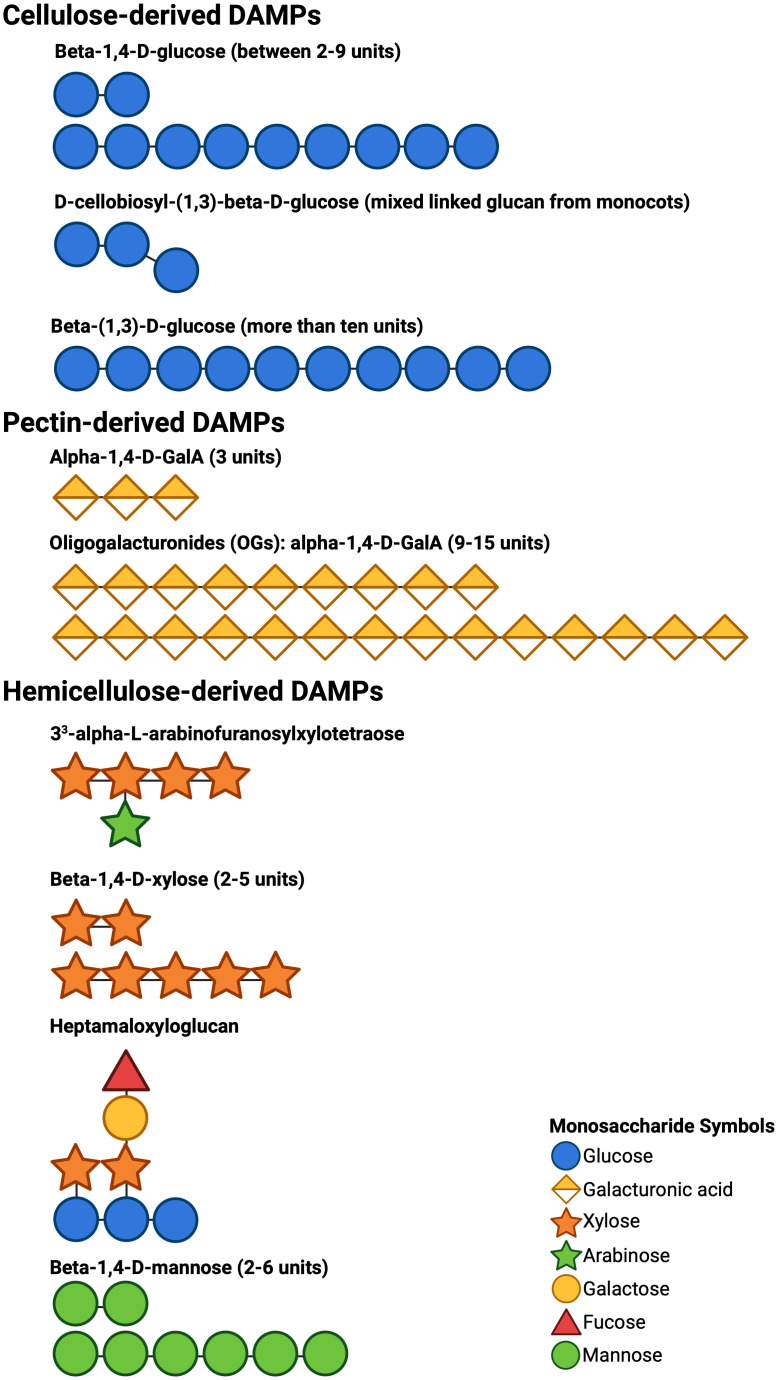
Cartoon representations of DAMP structures using the accepted symbols in glycobiology. The structures of DAMPs depicted were reported to be produced from the CW components cellulose, pectin, and hemicellulose by the action of CWDEs secreted by pathogens. These DAMPs are known to elicit PTI responses in various plant species (Molina et al., 2024).

CW-degrading enzymes (CWDEs) are one of the tools that pathogens use to breach the CW (Kubicek et al., 2014; Albersheim et al., 1969). They secrete a variety of CWDEs that enzymatically break down different CW polysaccharides, releasing the respective oligosaccharides (Wan et al., 2021, **Figure 1**). These CW fragments, called damage-associated molecular patterns (DAMPs), are perceived by the plant’s plasma membrane pattern recognition receptors (PRRs), initiating pattern-triggered immunity (PTI, Bacete et al., 2018). A variety of DAMPs have been identified that elicit a different defense responses in plants, including calcium signaling, reactive oxygen species (ROS) production, plant defense gene expression, MAPK phosphorylation, disease resistance, hormone accumulation, callose production, and phytoalexin production (Molina et al., 2024). The first DAMP identified was oligogalacturonides (OGs) derived from pectin (Hahn et al., 1981). Later, WAK1 was characterized and identified as a PRR that recognizes OGs and initiates PTI signaling in the cell (Decreux and Messiaen, 2005; Brutus et al., 2010). Since then, other CW components have been proposed to contribute to plant immunity (**Figure 1**, Molina et al., 2024; Swaminathan et al., 2022).

**Figure 2:**
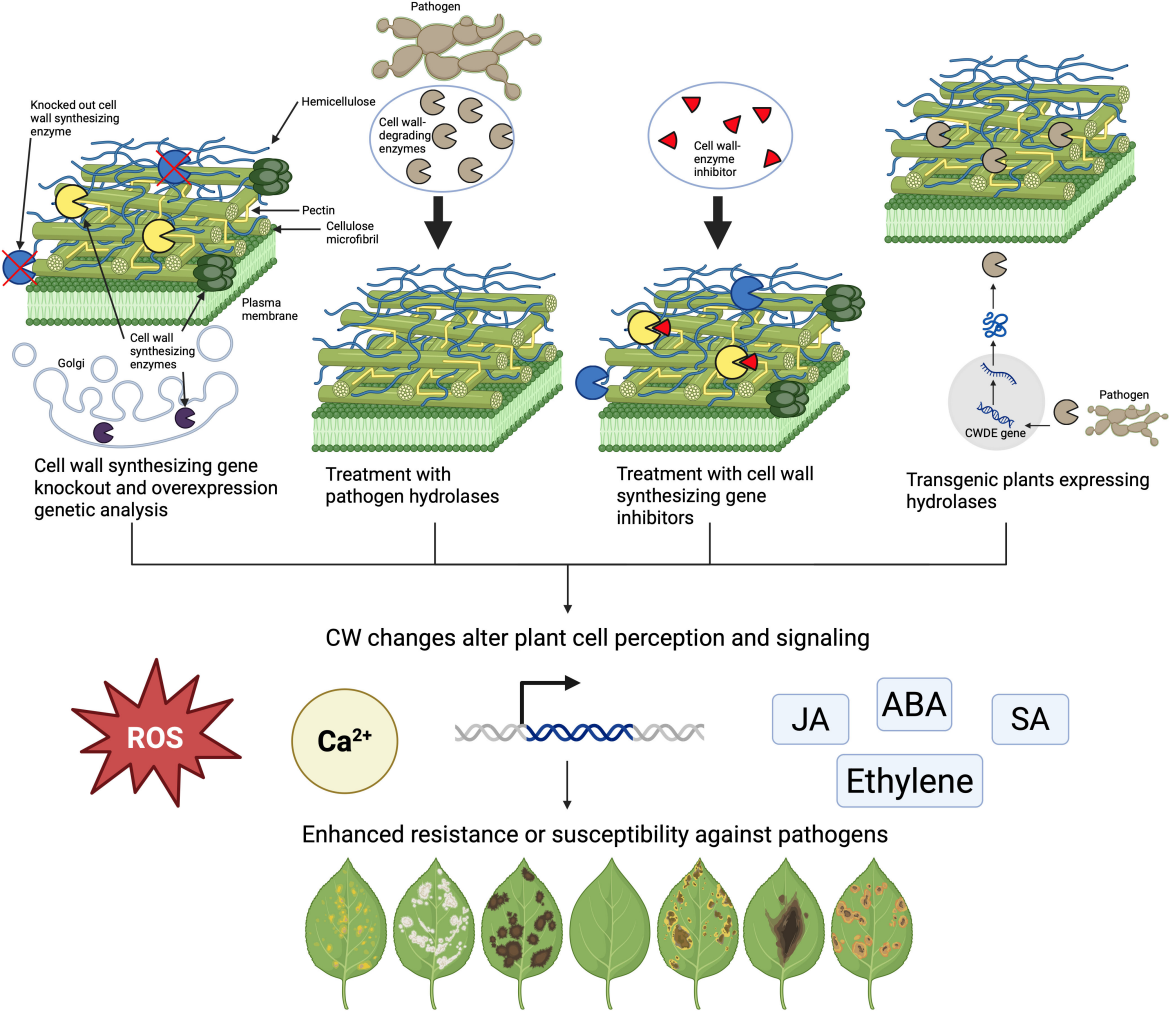
Four different approaches are commonly used to alter the CW to study its impact on plant immunity. Host plant genes involved in CW construction can be manipulated, CWs can be treated with isolated microbial enzymes, CW synthesizing enzyme inhibitors can be applied, or genes encoding CWDEs can be transformed into the plant. All these changes can have different effects on CW composition, plant morphology, hormone pathways, gene expression, reactive oxygen species (ROS) production, calcium signaling, and other PTI responses. These changes can alter how plants respond to pathogens.

The general approach used to understand how the CW contributes to plant immunity consists of making targeted changes to the CW and observing the alteration in plant immune signaling and the enhanced resistance or susceptibility of the plant to the pathogen (**Figure 2**). There have been a variety of strategies to make specific modifications to the CW composition and structure (**Figure 2**). The most common approach so far has been leveraging CW genetics by producing CW synthesizing mutants and corresponding overexpression lines (Molina et al., 2021). Another approach is to use CW enzyme inhibitors to alter the production of a specific CW component (Tateno et al., 2016) or to treat plants with extracted hydrolases isolated from pathogens (Sella et al., 2013). Finally, plants can be transformed to express CW-modifying enzymes so that CWs are altered post-synthetically (Ferrari et al., 2008). The CWs of these modified plants are analyzed and correlated to their overall response to the pathogen compared to control plants. The explanation for the observed resistance or susceptibility is done by verifying the underlying immune pathway using PTI responses such as defense gene expression analysis, hormone accumulation, callose production, MAPK phosphorylation, and ROS production (Swaminathan et al., 2022, **Figure 2**). This review will summarize the research and results obtained from each of these CW-modifying methods. Finally, advantages, disadvantages, and knowledge gaps that remain will be addressed.

## 2 Knocking out and overexpressing genes encoding CW-synthesizing enzymes

One of the most common approaches used to study the CW and its role in plant defense is through genetic manipulations (Farrokhi et al., 2006). It has repeatedly been shown that the absence or overexpression of a CW-synthesizing protein alters the CW structure and composition (Farrokhi et al., 2006). As a result, the plant has an altered response to pathogens. In some cases, the altered abundance of the enzyme causes the plant to be more resistant, while in other cases, the plant is more susceptible (Molina et al., 2021). Sometimes the transgenic/mutant plant is more resistant to one type of pathogen, but susceptible to others (Molina et al., 2021). The exact molecular mechanisms behind the resistance or susceptibility are not the same across different CW synthesizing mutants and overexpression lines. Frequently, the exact molecular mechanism behind the altered pathogen perception is obscure because many of the PRRs and their coreceptors are still uncharacterized and how CW integrity (CWI) pathways interact with pattern-triggered immunity is still an active area of research (Bacete and Hamann, 2020). Gene expression analysis, measurements of hormone accumulation, and other standard immune responses are used as an indicator of whether a plant immune pathway is impacted or if the mechanism is entirely independent. Previous studies have shown that cellulose, pectin, and hemicellulose have diverse contributions to plant immunity. The mutants for each major component of the CW and discoveries on their impacts to plant immunity are reviewed below and summarized in **Supplementary Table 1**.

### 2.1 Cellulose

Cellulose is synthesized by cellulose synthases (CESAs) that are found as a complex in the plasma membrane (Hernández-Blanco et al., 2007). The primary CW in Arabidopsis is synthesized by CESA1, CESA3, and CESA6, while the secondary CW in Arabidopsis is synthesized by CESA4, CESA7, and CESA8 (Hernández-Blanco et al., 2007). Mutations in these genes alter the CW composition and structure. The primary CW is produced early in plant development, and these CWs are flexible for future cell expansion (McFarlane, 2023). Secondary CWs are produced in specialized cells such as in tracheary elements to provide extra rigidity for water movement (McFarlane, 2023). Also, CWs may be strengthened with additional polysaccharides including cellulose during abiotic or biotic stress (Chowdhury et al., 2014). For example, barley leaves produce additional callose, cellulose, heteroxylans, phenolic compounds, and antimicrobial compounds at sites of *Blumeria graminis* f. sp. *hordei* penetration (Chowdhury et al., 2014).

*cev1* (Ellis et al., 2002) and *ectopic lignin* (*eli1*, Caño-Delgado et al., 2003) have mutations in the *CESA3* gene. Both mutants had less cellulose than control plants and *cev1* mutants accumulated more anthocyanin. *eli1* mutants compensated for the reduction in cellulose by producing lignin. *cesa3^mre1^* also has less cellulose and shorter, enlarged roots (Desaint et al., 2024). Secondary CW mutants typically have irregular xylem (irx) or a collapsed xylem phenotype due to CW weakening. *irx1* (mutation in *CESA8*) and *irx3* (mutation in *CESA7*) had less cellulose content (Brown et al., 2005). Although a triple *cesa7* mutant in wheat had no significant changes in plant morphology, it still had a reduction in cellulose compensated with lignin (Zhang et al., 2025). Other cellulose enzymes are important for synthesizing cellulose at papillae to strengthen the CW, which are made near appressoria where pathogen penetration occurs (Chowdhury et al., 2014; Douchkov et al., 2016). When the *Hordeum vulgare* (barley) *cellulose synthase D2* (*HvCslD2*) gene was silenced using RNA interference (RNAi), the plants produced less cellulose and more arabinoxylan in the epidermis. Additionally, the papillae produced by the plant during *B. graminis* infection had less cellulose (Douchkov et al., 2016). Overall, cellulose synthase mutants have less cellulose producing brittle CWs which is compensated for with lignin or arabinoxylan.

These cellulose-synthesizing mutants had various responses to pathogen infection, indicating the importance of the CW in determining plant immunity. The primary CW cellulose synthase mutant *cev1* was resistant to three different species of powdery mildew (Ellis and Turner, 2001). *CESA3* was a quantitative trait locus (QTL) in a genome-wide association study for Arabidopsis resistance to *Ralstonia solanacearum* under normal temperatures (27°C) and heat stress (30°C, Desaint et al., 2024). *eli1* and *cesa3^mre1^* were resistant to *R. solanacearum* GMI1000 at both 27°C and 30°C. Mutations in the other primary CW CESAs were important to *R. solanacearum* resistance as well. *cesa1^rsw1–10^* in Ws-2 and *cesa6^prc1–1^* in Col-0 are both resistant at 30°C compared wild-type control plants. Secondary CW Arabidopsis mutants *irx1* (*CESA8* mutant), *irx3* (*CESA7* mutant), and *irx5* (*CESA4* mutant) were all resistant to *R. solanacearum* and *Plectosphaerella cucumerina* (Hernández-Blanco et al., 2007). Similar results are seen across plant species. When three copies of *CESA7* were knockout in wheat, these plants were more resistant to *Puccinia striiformis* compared to wild-type, but when it was overexpressed, it was more susceptible (Zhang et al., 2025). However not all *CESA* genes are susceptibility factors. *CESAs* involved in papillae formation promote resistance. *Hordeum vulgare* plants that have silenced *HvCslD2* gene expression involved in papillae formation were more susceptible to *B. graminis* (Douchkov et al., 2016). Generally, mutations in primary and secondary CW CESAs make plants more resistant; however, mutations in cellulose synthases involved in CW strengthening make plants more susceptible to infection.

The immune mechanisms responsible for the observed susceptibility and resistance in these cellulose synthase mutants are also not consistent across all CESA mutants. Primary CW mutants have higher hormone accumulation and plant defense gene expression. *cev1* mutants constitutively accumulated the stress-responsive hormones jasmonic acid (JA) and ethylene (ET, Ellis et al., 2002). The extra lignin in *eli1* is produced by the upregulation of cinnamoyl-CoA reductase 2 (*CCR2*), which is expressed during plant defense responses (Caño-Delgado et al., 2003). The JA and ET defense genes *VSP1* and *PDF1.2* have enhanced expression in *cesa3^mre1^* compared to control plants (Desaint et al., 2024). However, altered root morphology is also responsible for increased resistance in this mutant because cut *cesa3^mre1^* and Col-0 roots were infected by *R. solanacearum* faster. However, *cesa3^mre1^* was still infected slower than Col-0 indicating that altered immune pathways are important (Desaint et al., 2024). Secondary CW mutants can have increase defense gene expression, but it appears that other hormones may also mediate the immune response. Secondary CW mutants *irx1, irx3*, and *irx5* crossed with various hormone mutants did not change resistance in these plants compared to wild-type indicating that the mechanism was independent of salicylic acid, jasmonic acid, and ethylene production (Hernández-Blanco et al., 2007). Instead, abscisic acid (ABA), a plant hormone not normally involved in plant immunity, appears to be important (Hernández-Blanco et al., 2007). ABA, however, does not appear to be important in other species such as wheat. The resistance in the secondary CW mutant *cesa7* in wheat involved more hydrogen peroxide production and defense gene expression including *TaPR1, TaPR2, TaPR5*, and other PTI marker genes. CESAs that strengthen the CW do not appear to have alterations in immune response. Plants that silence *HvCslD2* in *Hordeum vulgare* had normal phenolic compound accumulation compared to wild-type plants, a common defense response in barley and wheat (Douchkov et al., 2016). Instead, the CW composition of plants with reduced *HvCslD2* expression could induce susceptibility because its CW was degraded more easily by CWDEs compared to control plants. While resistance in primary and secondary CW CESA mutants is often mediated by characterized immune pathways and hormones, ABA also appears to mediate the resistance observed in some secondary CW mutants. Additionally, morphological changes in root architecture and CW composition also have important roles in determining plant responses to pathogens because they prevent pathogen access inside the plant cell and vasculature.

### 2.2 Pectin

Pectin consists of a group of complex polysaccharides, including homogalacturonan (HG), rhamnogalacturonan I (RG-I), and rhamnogalacturonan II (RG-II, Lampugnani et al., 2018; Delmer et al., 2024; Caffall and Mohnen, 2009). Pectin can be modified with methyl and acetyl groups (Lampugnani et al., 2018). Pectin is synthesized in the Golgi in a highly methyl-esterified form (Ibar and Orellana, 2007; Temple et al., 2022). Once it is secreted into the CW, endogenous plant CW-modifying enzymes like pectin methyl esterases (PMEs) can demethylate the pectin during plant physiological responses (Lionetti et al., 2012; Levesque-Tremblay et al., 2015; Jolie et al., 2010). The enzymatic activity of PMEs is inhibited by pectin methyl esterase inhibitors (PMEIs, Coculo and Lionetti, 2022). Pectin is acetylated by pectin acetyltransferases (Shahin et al., 2023) and HG can be broken down by polygalacturonases or pectate lyases (Lampugnani et al., 2018). Much research has been done on PMEs and PMEIs and their influence on pathogenicity, which will be the focus of this section. Other pectin mutants that have been linked to plant immunity will also be mentioned, though fewer details are available.

#### 2.2.1 Pectin methyl esterase

*pme* mutant plants are expected to have less pectin methyl esterase activity and, consequently, more methylated HG. As expected, *pme3* Arabidopsis mutants had less PME activity and more methylated HG compared to wild-type (Raiola et al., 2011). These results are similar in other plants; for example, *NaPME1* was silenced using RNAi in *Nicotiana attenuate* (Körner et al., 2009). Following treatment of pest *Manduca sexta* oral secretions, PME activity and methanol released as a byproduct of PME activity were reduced compared to the control (Körner et al., 2009). However, a larger-scale study exploring a variety of different PMEs in Arabidopsis found that only 12 of the screened *pme* mutants had altered CW structure based on Fourier transform infrared microspectroscopy (Bethke et al., 2014). Of the *pme* mutants that had altered CW structure and were susceptible to *Pseudomonas syringae* pv *maculicola* ES4326, none of them, including double, triple, and quadruple mutants, were found to have reduced PME activity during infection. There are 66 *PME* genes in Arabidopsis, and many may have redundant functions and not all of them are involved in plant immunity, which may explain why changes in PME activity were not detected (Bethke et al., 2014).

Reducing *PME* gene expression tends to make plants more resistant to pathogens. Arabidopsis *pme3* mutants were more resistant to *Botrytis cinerea* and *Pectobacterium carotovorum* compared to control plants (Raiola et al., 2011). This pattern stays consistent in *Nicotiana tabacum* where silenced *PME* gene expression prevented tobacco mosaic virus movement early during infection (Chen and Citovsky, 2003). Additionally, Arabidopsis overexpressing *PME3* were susceptible to *Heterodera schachtii* while *pme3* mutants were resistant (Hewezi et al., 2008). However, not all *pme* mutants were found to be resistant to all pathogens, including insect pests. *pme3*, *pme17*, *pme35*, *pme39*, *ppme1*, *pme31*, *pme44*, and various combinations of double, triple, and quadruple mutants were more susceptible to *Pseudomonas syringae* pv *maculicola* ES4326; however, none of the *pme* mutants tested in this study had altered resistance to *Alternaria brassicicola* compared to wild-type (Bethke et al., 2014). Also, *Maduca sexta* larvae performed better on *Nicotiana attenuata* plants with reduced *PME1* gene expression (Körner et al., 2009).

The mechanisms of the observed changes in plant response to pathogens are somewhat understood and seem to be largely independent of immune pathways. Reactive oxygen species (ROS) production and gene expression tied to both jasmonic acid and salicylic acid defense pathways, (*PR1*, *PDF1.2*, *WR3*, *JR1*, *PAD3*) was the same in both *pme3* mutants and wild-type (Raiola et al., 2011). While the large-scale *pme* mutant screen didn’t include testing of mutant lines for altered plant defense responses, it was found that PME activity in wild-type Arabidopsis was induced by microbe-associated molecular patterns (MAMPs), necrotrophic fungus *A. brassicicola*, and bacteria *Pseudomonas syringae* pv *maculicola* ES4326 (Bethke et al., 2014). This induction of PME was independent of salicylic acid and ethylene but required jasmonic acid (Bethke et al., 2014). This additional evidence indicates the importance of PMEs in the response to a wide variety of different pathogens and elicitors, however the mutant *PMEs* tested were not useful for understanding the role of PME during pathogenesis because PME activity could not be correlated with disease resistance (Bethke et al., 2014). In this case other approaches may be more useful. Patterns of PME activity and resistance may depend on plant species and infection style because *Nicotiana attenuata* with less *PME1* gene expression had less salicylic acid accumulation, and the absence of PME activity produced less methanol, which mediates the defense response (Körner et al., 2009; Ngouémazong et al., 2012).

#### 2.2.2 Pectin methyl esterase inhibitor

PMEI is an inhibitor of PME activity that helps determine the degree of pectin methylation and plays an important role in plant immunity. Plants with less PMEI are expected to have more PME activity and less methylated pectin while plants with more PMEI are expected to have less PME activity and more methylated pectin. Experiments reflect this anticipated pattern. Arabidopsis plants overexpressing *AtPMEI-1* or *AtPMEI-2* had reduced PME activity and more methylated pectin and Arabidopsis *pmei10*, *pmei11*, and *pmei12* mutants had increased PME activity and less methyl esterified pectin. Additionally, these mutants had less galacturonic acid and more pectin epitopes in hemicellulose-enriched CW fraction (Lionetti et al., 2017). Similar patterns are seen across species like *Gossypium hirsutum* (cotton) plants silencing *GhPMEI3* caused an increased PME activity (Liu et al., 2018). Additionally, an RNA sequencing experiment done on *Phaseolus vulgaris* identified *PvPMEI3* as the most upregulated CW remodeling enzyme compared to uninfected *P. vulgaris*. When it was silenced in Arabidopsis, PME activated increased 15% (de La Rubia et al., 2024). The connection between PMEI and the activity of PME appears to be consistent between different genes and across plant species.

The level of PMEI and PME activity correlated with disease resistance. PMEI-1 and PMEI-2 overexpression lines had smaller *B. cinerea* lesions compared to wild-type while Arabidopsis *pmei10*, *pmei11*, and *pmei12* had larger *B. cinerea* lesions (Lionetti et al., 2017). PMEIs were found to be important for disease resistance in other species. For example, *Capsicum annuum* (pepper) plants with reduced *CaPMEI1* via RNAi were more susceptible to virulent *Xanthomonas campestris* pv. *vesicatoria*, but not avirulent *Xanthomonas campestris* (An et al., 2008). Overexpressing the same protein in Arabidopsis conferred resistance to *Pseudomonas syringae* pv. *tomato* but not *Hyaloperonospora parasitica* (An et al., 2008). In addition, *Gossypium hirsutum* plants with reduced *GhPMEI3* gene expression were more susceptible to *Verticillium dahliae* (Liu et al., 2018). Interestingly, when this same gene was expressed in Arabidopsis, the plants had less pectin and were more resistant to *V. dahliae* (Liu et al., 2018). *pmei3* and *pmei3xpmei13* Arabidopsis mutants developed more chlorosis from *Pseudomonas syringae* infection than Col-0 wild-type plants (de La Rubia et al., 2024). PMEI is also important for defense against aphids since *pmei13* mutants are more susceptible to aphid *Myzus persicae* phloem access and sap drainage (Silva-Sanzana et al., 2019). Consistently, mutating *PMEI* decreases resistance to many different pathogens while overexpressing *PMEI* increases resistance.

The molecular mechanism for the resistance appears to be independent of normal immune pathways in Arabidopsis. *B. cinerea* grew more slowly on CWs extracted from PMEI-1 and PMEI-2 overexpression Arabidopsis lines compared to wild-type plants; however, the AtPMEI-1 or AtPMEI-2 of plants did not interact with the PME of *B. cinerea*, suggesting that there is no direct inhibition of pathogen CWDEs by the host PMEI (Lionetti et al., 2007). The *pmei10*, *pmei11*, and *pmei12* mutants produced hydrogen peroxide and callose normally compared to wild-type, so the defense pathways are not compromised in these plants (Lionetti et al., 2017). However, the mechanism may differ in other plant species. For instance, in *Capsicum annuum* plants with reduced *CaPMEI1* expression, defense gene expression was reduced. Also, CaPME11 inhibits PME and prevents *Fusarium oxysporum, A.brassicicola*, and *B. cinerea* growth (An et al., 2008). Similarly, in *Gossypium hirsutum*, GhPMEI3 interacts with PMEs, and *GhPMEI3* and *GhPME31* gene expression was altered after ethylene and jasmonic acid treatment (Liu et al., 2018). In *Phaseolus vulgaris*, the degree of methylesterification was hypothesized to cause susceptibility in the *pmei3* and *pmei3xpmei13* mutant Arabidopsis plants and moreover in *Phaseolus vulgaris* (de La Rubia et al., 2024). At least in Arabidopsis and cotton, it is hypothesized that if there are more PMEIs, then PMEs are prevented from demethylating the pectin, which becomes an additional barrier for pathogens to break down, slowing the process of infection (Lionetti et al., 2017; Liu et al., 2018). Pectin acetylation appears to have a similar mechanism because when citrus plants overexpress *CsPAE2* (pectin acetylesterase), the plants were more susceptible to citrus bacterial canker caused by *Xanthomonas citri* (Li et al., 2020). When *CsPAE2* expression was silenced in citrus plants, the plants were more resistant (Li et al., 2020).

Recent research has begun to understand the role of long noncoding RNAs (lncRNAs) in disease resistance (Zhang et al., 2022). lncRNAs are longer than 200 nucleotides and don’t have an open-reading frame. Two lncRNAs, *lncRNA2* and *lncRNA7* were found to target the two respective *Gossypium barbadense* cell wall modifying enzymes, *GbPG12* and *GbPMEI13*. When *PG12* gene expression was silenced, cotton plants were more resistant to *Verticillium dahliae*, however when *PMEI13* was silenced cotton plants were more susceptible. Additionally, Arabidopsis plants overexpressing *GbPMEI13* were more resistant to *V. dahliae* than wild-type plants. Overall, *lncRNA7* and *PG12* increase resistance to *V. dahliae*, while *lncRNA2* and *GbPG12* decrease resistance. The increase in methylated pectin can inhibit mycelial growth and spore germination. Oligogalacturonides were found to modulate the expression of these lncRNAs. OGs upregulated *lncRNA7* which increases PMEI13 gene expression while at the same time OGs downregulated *lncRNA2*, which also downregulates GbPG12. Overall, the modulation of pectin modifying enzymes via lncRNAs can regulate resistance to *V. dahliae* (Zhang et al., 2022).

#### 2.2.3 Other pectin-modifying enzymes

There are other important pectin-modifying enzymes that have been identified to confer resistance when mutated. The Arabidopsis *pmr6* and *pmr5* mutants were resistant to the powdery mildew species *Erysiphe cichoracearum* and *Erysiphe orontii* in a forward genetics screen (Vogel et al., 2002). However, these mutants were indistinguishable from wild-type when challenged with *Pseudomonas syringae*, *Peronospora parasitica*, *Plectosphaerella cucumerina*, *R. solanacearum*, and *B. cinerea* (Vogel et al., 2002, 2004; Hernández-Blanco et al., 2007). The *pmr6* plants have a mutation in a pectate lyase gene that is an endogenous gene that degrades pectin (Vogel et al., 2004). *pmr5* has a mutation in a pectin acetyltransferase gene, which adds galacturonic acid residues to HG (Chiniquy et al., 2019). The *pmr6* mutants had more pectin, and *pmr5* plants had less pectin and less pectin that is methyl esterified or acetylated (Vogel et al., 2002). The mechanism of resistance in both these mutants was independent of SA- and JA-mediated defense pathways because *PR1* and *PDF1.2* gene expression was unaltered compared to wild-type (Vogel et al., 2002, 2004). Additionally, *pmr5* resistance was not dependent on *PAD3* or *WAK1* pattern recognition receptor gene expression. These results suggest that resistance could be mediated by a loss of a susceptibility factor or the activation of an unknown defense pathway (Vogel et al., 2004).

Polygalacturonase (PG), like pectate lyase, degrades pectin in the cell wall and was found to have important implications in fruit ripening and fruit resistance to fungal decay (Paniagua et al., 2022). When the polygalacturonase *FaPG1* was silenced in *Fragaria ananassa*, strawberry fruits lost less weight had firmer fruit, and experienced less fungal decay after harvesting compared to control strawberries (Paniagua et al., 2022). Although not yet tested, it is predicted that the loss of *FaPG1* altered the content of OGs in the CW which are responsible for the decreased susceptibility to fungal decay after harvesting (Paniagua et al., 2022).

Polygalacturonases also interact with expansins (EXP) and together are important in the processes of fruit ripening and fruit susceptibility to pathogen infection in tomato (Cantu et al., 2008). Expansins are CW proteins with no catalytic activity that aid in CW loosening and expansion by disrupting noncovalent bonds between polysaccharides (Cosgrove, 2024). When *LePG* and *LeEXP1* were both silenced in tomato, the pectin became less soluble, and the enrichment of water-soluble rhamnose and arabinose during the ripening process was absent. Additionally, tomatoes with reduced *LePG* and *LeEXP* expression were firmer during ripening and more resistant to *B. cinerea*. However, silencing just one of these genes did not alter either its firmness or susceptibility (Cantu et al., 2008).

Enzymes that make precursors for pectin synthesis were also shown to mediate plant immunity. UDP-D-glucuronate 4-epimerase (GAE) interconverts UDP-D-GlcA and UDP-D-GalA, which is a precursor required for pectin synthesis (Bethke et al., 2016). The CWs of Arabidopsis *gae6* and *gae1gae6* mutants had less GalA, but more neutral sugars and cellulose compared to wild-type. Additionally, *gae1gae6* CWs had less HG but the same degree of pectin methyl esterification. The leaves in the mutants were brittle and had more ion leakage compared to wild-type. The *gae6* and *gae1gae6* mutants were susceptible to *Pseudomonas syringae* pv *maculicola* ES4326 and two different isolates of *B. cinerea*. However, normal defense responses were unchanged. Camalexin production, hydrogen peroxide accumulation, *PAD3*, *SID2*, and *PR1* expression were indistinguishable from Col-0. Callose deposition was reduced in *gae1gae6* plants, which could not be confirmed as the cause for its susceptibility to *B. cinerea*. It was suggested that the reduction in pectin, and thus the reduction in OG elicitor production was responsible for compromising callose production and immunity in *gae1gae6* (Bethke et al., 2016).

Pectin synthesis can also be modulated when lignin synthesis is disrupted. When lignin biosynthesizing enzymes hydroxycinnamoyl transferase (HCT) and cinnamoyl CoA reductase (CRR) gene expression were altered, many *PR* defense genes were upregulated, lignin content was reduced, and cell wall remodeling genes were overexpressed (Gallego-Giraldo et al., 2020). Both altered lines had more abundant rhamnogalacturonan, arabinogalactan, xylan, pectin, and xyloglucan epitopes in the oxalate and carbonate CW extracts compared to the 4M KOH CW extract. More OG elicitors were also found in HCT and CRR altered plants. A *Caldicellulosiruptor bescii* strain lacking functional pectinase genes was able to grow more on the HCT-RNAi *ccr1* plants compared to wild-type plants, indicating that the increased expression of pectin modifying enzymes allowed the CW to be digested by pectinase-deficient *C. bescii*. When Arabidopsis Dehiscence Zone Polygalacturonase 1 (ADPG1) gene expression was also eliminated in the *ccr1* and HCT-RNAi lines, OG production and *PR1* gene expression was reduced to wild-type levels, indicating that ADPG1 is necessary for both of these defense responses. Overall, the loss of normal lignin production was proposed to be perceived by CW integrity sensors which altered the expression of CW modifying enzymes, producing DAMPs and activating the *PR* genes (Gallego-Giraldo et al., 2020).

### 2.3 Hemicellulose

Plant CWs are composed of a diversity of hemicellulose polysaccharides, including xyloglucans, xylans, and mannans (Scheller and Ulvskov, 2010). Xyloglucan has a beta-1,4-glucan backbone with xylose, galactose, and fucose side changes (Scheller and Ulvskov, 2010). Xylan is a beta-1,4-linked xylosyl backbone with arabinose and glucuronic acid side chains, which can be methylated or feruloylated (Scheller and Ulvskov, 2010). Mannans are less common but contain a beta-1,4-linked mannose chain (Scheller and Ulvskov, 2010). Hemicellulose mutants can be loosely grouped by hemicellulose-synthesizing proteins, hemicellulose hydrolyzing/modifying enzymes, and finally, hemicellulose acetylating enzymes.

#### 2.3.1 Hemicellulose-synthesizing proteins

The hemicellulose xylan is synthesized by multiple xylosyltransferases. A homologue of *ZmXYXT1* called *ZmXYXT2* was identified as a quantitative trait locus (QTL) linked with maize resistance to *Fusarium verticillioides* (Xu et al., 2025). The *zmxyxt2* mutant plants had thinner CWs and had less hemicellulose (approximately 22% decrease) and less arabinose (63% decrease), xylose (57% decrease), glucose (59% decrease), galactose (55% decrease), galacturonic acid (56% decrease), and less ferulic acid (statistically significant but numbers not reported). Other than the leaf sheaths and blades being more brittle, there were no differences in plant height, stem diameter, ear height, or grain yield in the mutant. Maize plants overexpressing *ZmXYXT2* had more hemicellulose (24% increase) and statistically more glucose, xylose, arabinose, and ferulic acid (exact percentages were not reported). Similar to the mutant, the overexpression line did not have any major phenotypes. The *zmxyxt2* mutants were susceptible to *Fusarium verticillioides* while ZmXYXT2 overexpression lines were more resistant. The expression of the plant defense genes *ZmPR7, ZmPR6, ZmPR4*, and *ZmCRA1* was higher and faster in *xmxyxt2* mutant, indicating that the susceptibility of *xmxyxt2* may be related to a weaker CW. The reduction in xylan and ferulic acid can impair crosslinking and CW thickness (Xu et al., 2025).

Another type of xylan, arabinoxylan, is also synthesized in addition to cellulose and callose to form papillae to strengthen the plant CW, where pathogen appressoria form (Chowdhury et al., 2017). A variety of arabinoxylan-synthesizing genes were transiently overexpressed and silenced in barley. When *GT43*, *GT61*, and *GT31* were silenced, fungal penetration was not prevented. When *GT75*, *GT8*, *GT61*, and *GT47* were transiently overexpressed, fungal penetration was prevented. However, transient overexpression of *GT47* and *GT6* increased powdery mildew penetration. The combination of GT43 and GT47 induced resistance in barley to powdery mildew, suggesting that the combination of multiple enzymes is important because of how they complex together in the epidermis (Chowdhury et al., 2017). This evidence supports the idea that *GT43* and *GT47* may interact together to synthesize the xylan backbone in papillae. The GTs responsible for the synthesis of heteroxylan often have redundant functions, which makes identifying important GTs for resistance challenging as different genes may compensate for the loss or overexpression of one GT (Chowdhury et al., 2017).

#### 2.3.2 Hemicellulose-modifying and hydrolyzing enzymes

There are many different hemicellulose-modifying enzymes that target specific types of hemicelluloses. Xyloglucan endotransglycosylase/hydrolases (XTHs) are important for hydrolyzing and rejoining xyloglucan strands and are necessary for cell expansion, fruit ripening, abscission, and differentiation (Flors et al., 2007; Niraula et al., 2021). *Glycine max* (soybean) plants with reduced *XTH43* gene expression via RNAi did not change monosaccharide composition compared to wild-type, but it had longer xyloglucan chains that were less tightly bound (Niraula et al., 2021). On the other hand, soybean plants that were overexpressing *XTH43* had more tightly bound xyloglucan. Soybean roots with silenced *XTH43* had more severe cyst infection from *Heterodera glycines*, while overexpression plants were more resistant (Niraula et al., 2021). When *XTH43* gene expression is increased, xyloglucan lengths are longer, which is thought to prevent the CW expansion required for *Heterodera glycines* to produce a feeding structure aiding parasitism.

Beta-1,4-endoglucanases (EGases) also hydrolyze and rejoin xyloglucan strands like XTHs. *Cel1* is an EGase that was previously found to be correlated with fruit ripening and downregulated during *Botrytis cinerea* infection. Plants with reduced *Cel1* and *Cel2* gene expression were more resistant to *B. cinerea*, but more susceptible to *P. syringae* (Flors et al., 2007). No change in susceptibility was observed in the fruits against *B. cinerea* compared to wildtype. This resistance was correlated with increased *PR1* and *LoxD* gene expression involving both the SA and JA hormone pathways. Arabidopsis plants with silenced EGases *Cel1* and *Cel2* had more callose in the epidermis after *B. cinerea* infection and resistance in the mutant was lost if callose synthesis was inhibited (Flors et al., 2007). Overall, it was hypothesized that the absence of EGase activity was perceived by the plant, activating defense responses and producing more callose in a shorter amount of time (Flors et al., 2007).

Another enzyme, beta-D-xylosidase (BXL), has predicted glycosyl hydrolase domains that target xylans and arabinans (Guzha et al., 2022). BXL1 has been shown to have xylosidase activity in the CW and is expressed in tissues synthesizing the secondary CW (Goujon et al., 2003). BXL4 has similar enzymatic activity as BXL1 since it can complement *bxl1* seed coat mutant phenotype when overexpressed. Arabidopsis *bxl4* mutant plants had the abundance of HG and RG to wild-type, except with more arabinose and arabinosyl residues (Guzha et al., 2022). *bxl4* mutants didn’t have any growth defects (Guzha et al., 2022). The *bxl4* mutants were susceptible to *B. cinerea*, while Arabidopsis overexpressing BXL4 were more resistant (Guzha et al., 2022). *bxl4* mutant plants were unable to accumulate as much JA-Ile and camalexin after infection, and JA markers were not upregulated as much as wild-type plants after wounding (Guzha et al., 2022). *BXL4* overexpression in Arabidopsis had more *PDF1.2* and *PAD3* defense gene expression compared to the wild-type control (Guzha et al., 2022). Resistance could be a result of trimming arabinan side chains to allow more CW crosslinking, preventing fungal infection in combination with perception of altered CW composition or alternatively xyloglucan-derived DAMPs can also be produced inducing plant defense responses (Guzha et al., 2022).

BXL4 has also been found to contribute to the long-term, whole plant immune response called systemic acquired response (SAR, Bauer et al., 2023). BXL4 protein accumulates in the apoplast in plants during SAR. *bxl4* had unaltered initial local responses to *Pseudomonas syringae* compared to wildtype. However, long distance signaling via phloem and volatiles in *bxl4* was impaired. How BXL4 helps mediate SAR long distance signaling is still new, but fucose accumulation may be a contributing factor (Bauer et al., 2023).

#### 2.3.3 Hemicellulose-acetylating enzymes

Hemicellulose, like pectin, can be acetylated, which affects various properties of the CW (Gao et al., 2017). Many different proteins are found to be involved in acetylation: Reduced Wall Acetylation (RWA), Trichome Birefringence-Like (TBL), Altered Xyloglucan 9 (AXY9), and GDSL-type esterase/lipase proteins (GELP) (Gao et al., 2017; Rastogi et al., 2024). Arabidopsis *rwa2* mutant plants had less CW acetate and less O-acetylated fucosylated xyloglucan (Manabe et al., 2011). The *rwa2* mutants had an unaltered monosaccharide composition and phenotype compared to wild-type plants (Manabe et al., 2011). *Os*TBL1 and *Os*TBL2 are enzymes needed in rice for xylan monoacetylation (Gao et al., 2017). Rice *ostbl1* and *ostbl2* mutant plants had stunted growth and fewer acetyl esters (Gao et al., 2017). The overexpression lines had an unaltered phenotype but had more acetyl esters in the CW (Gao et al., 2017). The *esk1* mutant (a mutant in the TBL family) was identified from a suppressor screen that restores resistance to *PcBMM* of *agb1–2* mutants that were deficient in the heterotrimeric G-protein complex and unable to communicate PTI response downstream of pattern recognition receptors. The *esk1* mutants were stunted with excess branching (Escudero et al., 2017). Arabidopsis overexpressing *At*GELP53 had more esterase activity and less CW acetyl content compared to wild-type control while *atglep53* Arabidopsis mutants had less esterase activity and more CW acetyl content (Rastogi et al., 2024). The relationship between CW acetyl content and esterase activity are consistent across different mutant lines and species.

Altering the expression levels of these hemicellulose-modifying enzyme genes consequently alters their response to pathogens. Rice *ostbl1* and *tbl1tbl2* double mutants were more susceptible to leaf blight; however, the exact mechanism is untested (Gao et al., 2017). The *rwa2* mutant plants were resistant to *B. cinerea* but responded the same way as wild-type when challenged with the powdery mildew *Golovinomyces cichoracearum* (Manabe et al., 2011). The mechanism appears independent of normal immune pathways because the expression of *PDF1.2* and *PAD3* defense genes was unchanged compared to wild-type. The *agb1–2 esk1–7* double mutant plants were resistant to *PcBMM* compared to *agb1–2* plants; however, they remained susceptible to *P. syringae* pv. *tomato DC3000* and *Hyaloperospora arabidopsidis* (Escudero et al., 2017). While reduced ROS production and activation of *CYP81F2* and *WRKY33* defense genes in *agb1–2* was not restored by *esk1-7*, MAPK phosphorylation was restored by *esk1-7*. Additionally, ABA-related genes were constitutively overexpressed in *agb1–2 esk1–7* and *esk1-7*, but none of the SA, ethylene, or JA-related defense genes were upregulated. Arabidopsis overexpressing *At*GELP53 were resistant to *Pseudomonas syringae* and *Ralstonia solanacearum* potentially because the CW was more resistant to digestion or because DAMPs may be produced inducing a defense response (Rastogi et al., 2024). While hemicellulose modification-mediated resistance involved PTI, it appears that hemicellulose acetylation-mediated resistance involves a distinct, pathway.

#### 2.3.4 General trends

Recently, a large-scale approach used a panel of 34 CW mutants of a variety of different CW components, some of which were previously analyzed, to synthesize what is generally understood and confirm the patterns observed previously (Molina et al., 2021). The CW composition, resistance to three different pathogens (*Plectosphaerella cucumerina, Ralstonia pseudosolanacearum*, and *Hyaloperospora arabidopsidis*), and fitness tradeoffs were analyzed and correlated together to find causative trends linked to the CW composition (Molina et al., 2021). 29 CW mutants responded differently to the pathogens compared to wild-type, and most of these were more resistant to several pathogens with different infection styles, confirming the importance of the CW in plant immunity. These CW mutants tended to have less biomass and less seed production compared to wild-type. These phenotypes negatively correlated with resistance to two of the pathogens, suggesting a fitness trade-off. Glycome profiling results from a subset of the CW mutants indicated that the abundance of CW epitopes was significantly different in every mutant. Analysis correlating the CW results, the phenotypes, and disease resistance showed that an epitope in RGI and arabinogalactan correlated with the biomass phenotype, while galactomannans and acetylated mannans correlated with seed yield. Also, fucosylated-xyloglucans correlated with resistance to *P. cucumerina*, an undefined RGI epitope correlated with resistance to *R. psedosolanacearum*, and galactomannans and fucosylated xyloglucans correlated with resistance to *H. arabidopsidis*. When defense gene expression was analyzed, no relationship was found between defense gene expression and disease resistance, suggesting the presence of an alternative mechanism underlying the resistance of these mutants (Molina et al., 2021).

Additional mutants have been made in other CW components, such as expansins (Narváez-Barragán et al., 2020), callose (Chen et al., 2024; Voigt, 2014), lignin (Yadav and Chattopadhyay, 2023), and AGPs and other proteins (Rashid, 2016), which are outside the scope of this review but are discussed elsewhere.

## 3 Exogenous treatment with CW-synthesizing enzyme inhibitors and hydrolases

Another method for studying how the CW affects plant immune signaling and responses is to treat CWs with enzyme inhibitors or isolated CW-degrading enzymes (Tateno et al., 2016). Although this method is much less common, it has further complemented results observed when using gene knockout and overexpression lines, clarified the relationship between resistance and CW composition, and characterized damage-associated molecular patterns.

The CW cellulose synthase *cev1* mutant discussed earlier in this review had anthocyanin accumulation, more root hairs, and a shortened hypocotyl when grown in the dark (Ellis et al., 2002). To confirm that the phenotype observed in the mutant is due to a defect in cellulose synthesis, wild-type Arabidopsis plants were treated with 2,6-dichlorobenzonitrile (DCB) and isoxaben (IXB), which are cellulose biosynthesis inhibitors (Ellis et al., 2002). The treated plants had stunted leaves, anthocyanin accumulation, and increased gene expression of the plant defense genes *PDF1.2* and *VSP*. These results confirmed that cellulose synthesis inhibition causes the same phenotypes observed in the *cev1* mutant and that plant defense gene expression is also altered (Ellis et al., 2002).

A similar method was used to confirm the role of callose in pathogen resistance. Using RNAi, Arabidopsis with reduced hemicellulose-modifying enzyme gene expression *Cel1* and *Cel2* beta-1,4-endoglucanases (EGases) had more callose production in the epidermis after infection and were more resistant to *B. cinerea* (Flors et al., 2007). To test whether callose was responsible for this resistance, the RNAi plants were treated with 2-deoxy-D-glucose, which is an inhibitor of callose synthesis. RNAi plants treated with the inhibitor lost their enhanced resistance to *B. cinerea* compared to wild-type, suggesting that callose has a significant role in mediating the defense response (Flors et al., 2007).

Sometimes, a direct correlation between CW traits and plant resistance is not immediately clear, and treatment with hydrolases can clarify this relationship. Carrot cultivars were scored for disease susceptibility to *Mycocentrospora acerina*, and the CW compositions for each variety were determined with the goal of correlating resistance to CW traits (LeCam et al., 1994). The CW monosaccharide composition did not correlate with the resistance of the carrot varieties. However, correlations between the CW and resistance became clearer when carrot tissue was treated with enzymes (mostly polygalacturonases) extracted from *Mycocentrospora acerina*. Resistant carrot varieties had less tissue maceration compared to susceptible carrot varieties. A similar experiment was repeated on extracted pectin samples, and the solubilization rate was higher for susceptible carrot varieties than for resistant varieties (LeCam et al., 1994). Overall, it appeared that the pectin esterification and solubility were important for determining carrot resistance to *M. acerina*.

A similar approach was used in a different plant-pathogen system with similar results. *Hordeum vulgare* plants with silenced *HvCslD2* were more susceptible to *B. graminis*, but phenolic compound accumulation was normal (Douchkov et al., 2016). The CWs extracted from these plants treated with fungal cellulases, hemicellulases, and pectinases were digested faster than wild-type CWs, which may explain why the plants are susceptible even though the immune pathways remain uncompromised (Douchkov et al., 2016).

Enzyme treatments have also been used to characterize damage-associated molecular patterns (DAMPs) of susceptible and resistant plant varieties that are perceived by the plant and initiate defense responses. CWs of resistant and susceptible beans were treated with pathogen-secreted endopolygalacturonases, and the resulting pectin fragments were characterized and applied onto plants to test their effect on *PR* gene expression, ethylene hormone signaling, and other immune responses (Boudart et al., 1998). Galactose, arabinose, and rhamnose content was higher in the resistant CW fractions, while galacturonic acid was lower compared to the susceptible bean. Overall, resistant fragments had more branched rhamnogalacturonan. When resistant plants were treated with resistant fragments, beta-1,3-glucanase, chitinase, and ethylene activity increased. When the susceptible plants were treated with the susceptible fragments, chitinase and beta-1,3-glucanase did not increase as much, and there was no ethylene accumulation. When the susceptible plants were treated with resistant CW fragments, defense responses included increased beta-1,3-glucanase, chitinase, and ethylene activity. When the resistant plants were treated with susceptible CW fragments, glucanase, chitinase, and ethylene activities only weakly increased. Overall, the fragments produced by endoPG activity of resistant CWs initiated larger defense responses than fragments of CWs from susceptible plants (Boudart et al., 1998).

Similar experiments to determine DAMPs have been done in other species like tomato plants (Jiménez-Maldonado et al., 2018). Rhamnogalacturonan I (RG-I) isolated from potato was treated with rhamnogalacturonan lyase from *Cellvibrio japonicus*. When these treated RG-I fragments were applied onto tomato plants, beta-1,3-glucanase activity increased significantly 6 hours after treatment compared to control mock inoculated plants, but no significant differences were observed 0.5, 1, 24, or 28 hours after inoculation. Similarly, chitinase and peroxidase activity increased significantly 0.5 and 48 hours after treatment, but not 1, 6, or 24 hours after treatment (Jiménez-Maldonado et al., 2018). Treating polysaccharides with CWDEs and then applying them on plants can be helpful to characterize potential elicitors of plant immunity.

## 4 Transgenic plants expressing CWDE-encoding genes

Another approach that has been used to understand the relationship between the CW and plant immunity is by transforming plants to express hydrolases sourced from other organisms to modify the plant CW post-synthetically (Ferrari et al., 2008). Here, we group results that were obtained by studies using plants expressing CWDEs from pathogens and CW-modifying enzymes from other plant species.

### 4.1 Transgenic plants expressing pathogen CWDEs

Polygalacturonases (PGs) are enzymes that break down the pectin HG (Ferrari et al., 2006). Transgenic tobacco and Arabidopsis were generated to express PGII from *Aspergillus niger*. The transgenic plants were small, and the leaves were curled; however, this phenotype was complemented by co-expressing with *PvPGIP2* in tobacco. There was also less HGA in the transgenic tobacco and Arabidopsis. Transgenic tobacco and Arabidopsis were more resistant to *B. cinerea* and *P. syringae* pv. *tabaci*, however, Arabidopsis expressing an inactive PGII did not have enhanced resistance compared to wild-type. Additionally, plants expressing PGIP2 were more resistant, but had water-soaked lesions. Hydrogen peroxide accumulated in the PGII-expressing tobacco plants, but not in wild-type or PGIP2-expressing plants. The expression of the plant defense genes *POX* and *EAS1/2* was elevated compared to wild-type before and during *B. cinerea* infection. These defense genes were also expressed during oligogalacturonide (OG) treatment, so the resistance observed in the transgenic plants may be caused by an accumulation of this DAMP. Previously, it was suggested that OGs can decrease auxin activity in the plant, and it was found that treating PG transgenic plants with auxin made them susceptible to *B. cinerea* like wild-type plants. However, auxin treatment of wild-type plants did not alter their susceptibility to *B. cinerea*. It was found that IAA can induce rooting in wild-type tobacco, but higher concentrations of IAA were required to induce rooting in the PGII-expressing transgenic tobacco. Auxin degradation was unaffected in PG transgenic plants, so it appears that auxin signaling has been changed, which also may be involved in the enhanced resistance (Ferrari et al., 2006).

Tall fescue (*Schedonorus arundinaceus*) and perennial ryegrass (*Lolium perenne*) were transformed to express a vacuole-localized and apoplast-localized *Aspergillus niger* ferulic acid esterase (*FAEA*) (Buanafina and Fescemyer, 2012). The transgenic plants had increased FAEA expression, increased enzyme activity in the apoplast, fewer ferulate monomers, ferulic acid, and p-coumaric acid in their CWs. When treated with *T. reesei* cellulase, the transgenic plants released more reduced sugars than control plants. Larvae that fed on FAEA-expressing plants had a lower mortality rate and a shorter larval stage than larvae feeding on control plants. Overall, FAEA transgenic tall fescue CWs were more digestible by cellulase which allowed increased larval survival and growth because they are easily digested and assimilated (Buanafina and Fescemyer, 2012).

*Aspergillus nidulans* acetyl xylan esterase (*An*AXE) and rhamnogalacturonan acetyl esterase (*An*RAE) genes were transformed into Arabidopsis and *Brachypodium* with a signal peptide to secrete the enzyme into the apoplast (Pogorelko et al., 2013). The transgenic plants had more acetyl esterase activity and fewer acetyl groups in the CWs. The monosaccharide composition was unchanged in these plants. The transgenic Arabidopsis plants were more resistant to *B. cinerea* infection but performed similarly to wild-type when challenged with the bacteria *Pseudomonas syringae* pv *tomato* DC3000. The transgenic *Brachypodium* plants infected with the bacteria *Bipolaris sorokiniana* had smaller lesions compared to wild-type, but this resistance was not observed when challenged with *Xanthomonas oryzae*. Several plant defense genes were upregulated in uninfected transgenic Arabidopsis (*PAD3*, *WRK*, *CYP*, *PR5*, *bG2*) and *Brachypodium* (*PR5* and *bG2*). AnRAE-expressing Arabidopsis plants had more callose when compared to wild-type plants before infection. Additionally, hydrogen peroxide accumulation was significantly higher in Arabidopsis expressing AnRAE and AnAXE in comparison with wild-type (Pogorelko et al., 2013).

*A. nidulans* feruloyl esterase (AnFAE) was expressed in Arabidopsis and *Brachypodium* plants’ apoplasts (Reem et al., 2016). The apoplastic fluids in the transgenic plants had more feruloyl esterase activity compared to wild-type, and less ferulic acids were found in their CWs. Transgenic *Brachypodium* had more xylose and less glucose monosaccharides, and transgenic Arabidopsis monosaccharides were unchanged compared to wild-type. Arabidopsis transgenic plants released more reducing sugars when treated with xylanases and cellulases compared to wild-type, but the combined pectin methyl esterase and polygalacturonase treatment produced the same amount of reducing sugars compared to wild-type. *Brachypodium* transgenic plants released more reducing sugars after cellulase treatment only, but not after xylanase, pectin methyl esterase, or polygalacturonase treatment. The *AnFAE*-expressing Arabidopsis and *Brachypodium* plants were more susceptible to *B. cinerea* and *B. sorokiniana* infection, respectively. Several plant defense genes had significantly higher expression in AnFAE-expressing Arabidopsis during pathogen infection compared to wild-type, including *PGIP1*, *bG2*, and *WRKY40*, but *PR1* was downregulated. Some defense genes were also upregulated in *Brachypodium*, including *WRKY40*, *WR3*, and *RetOX*. The AnFAE-expressing plants had more crosslinked extensin; however, there was less HG-soluble protein and hydroxyproline. Basal expression of plant defense genes in both Arabidopsis and *Brachypodium*, unchallenged with pathogens, was unchanged compared to wild-type (Reem et al., 2016).

The global transcriptome and metabolome analyses were performed using Arabidopsis plants expressing AnAXE, AnRAE, and AnFAE (Reem et al., 2018). Differentially expressed genes in the transgenic plants included a diversity of gene functions, among which were transcription factors, stress response, and CW-related genes. Similar genes and pathways were shared in the resistant AnAXE- and AnRAE-expressing plants, while AnFAE-expressing plants had distinct differentially expressed genes and pathways. In the susceptible AnFAE expressing Arabidopsis plants, JA-responsive genes, WRKY transcription factors, and extensin-related genes were downregulated. On the other hand, resistant AnAXE and AnRAE plants had upregulated defense genes, like *PDF1.2b* and *PDF1.4* (Reem et al., 2018).

*A. nidulans* xylan acetyl esterase (AnAXE), rhamnogalacturonan acetyl esterase (AnRAE), and feruloyl esterase (AnFAE) were co-expressed in different combinations in Arabidopsis (Swaminathan et al., 2021). Plants expressing one or both AnAXE and AnRAE had reduced acetyl levels, while acetyl levels were unchanged in AnFAE-expressing plants. Feruloylation was reduced in all plants expressing AnFAE, even in combination with AnRAE or AnAXE. However, feruloylation was not altered in transgenic plants expressing only AnRAE, AnAXE, or both. Plants expressing AnFAE were more susceptible to *B. cinerea*, while plants expressing AnAXE or AnRAE were more resistant. Plants co-expressing two acetyl esterases, AnAXE and AnRAE, had a synergistic effect on their resistance when compared with plants expressing single enzymes. Co-expression of AnAXE or AnRAE with AnFAE compensated for the susceptibility of FAE-expressing plants, restoring their resistance to the level of wild-type plants. The expression of defense genes *WRKY*, *JR1*, and *PDF1.2* was reduced in all three single transgenic lines. AnRAE-expressing plants had higher *PAD3*, *RetOx*, and *PR1* gene expression. The AnAXE-expressing plants had higher *PR1* gene expression. None of the defense genes were upregulated in AnFAE-expressing Arabidopsis. Plants co-expressing different combinations of these enzymes had interesting gene expression patterns. For example, *PAD3*, *RetOx*, *PR1*, and *PDF1.2* were upregulated in the double transgenic plants AnAXE/AnRAE and AnRAE/AnFAE. Additionally, there was more hydrogen peroxide accumulation in all the double transgenic plants and in plants only expressing AnRAE. Overall, expressing a combination of CWDEs caused a synergistic increase in plant defense genes, particularly in *PDF1.2* (Swaminathan et al., 2021).

*Aspergillus nidulans* pectin methyl esterase (PME) was expressed in Arabidopsis plants, and these transgenic plants had shorter roots, smaller leaves, siliques, flowers, and stomata, likely caused by reduced cell expansion (Reem et al., 2020). Methyl esterification in the transgenic plants was reduced. When CWs were treated with polygalacturonases and pectin methyl esterase, the transgenic CWs in all the lines had fewer reducing sugars released. However, when treated with just polygalacturonases, more reducing sugars were released. Additionally, the transgenic plants had less galacturonic acid and more arabinose. Roots of *AnPME*-expressing plants were less susceptible to salt or osmotic stress in comparison with wild-type plants. While the exact mechanism for salt and osmotic stress resistance were not determined for these transgenic plants, it was hypothesized that increased calcium-mediated crosslinking allowed better water retention. One independent transgenic line was more susceptible to *B. cinerea* infection, while the other two lines were just as susceptible as wildtype control plants. The variation in response to *B. cinerea* was suggested to be caused by enhanced immune responses instead of weaker CWs because although all three independent lines had CWs that were more degradable by polygalacturonases, this did not correlate with increased *B. cinerea* susceptibility. This is supported by the observed increased ROS accumulation in the transgenic plants compared to wild-type. Expression of *PMEI10*, *PMEI11*, beta-glucanase 2, cytochrome P450, *EDS1*, *JR1*, *PAD3*, *PAD4*, *PDF1.2*, *PR1*, *PR5*, *WR3*, and *WRKY40* genes was elevated in unchallenged-PME expressing plants compared with wild-type. There were differences in how much these genes were expressed between the different lines, which may explain the different responses to *B. cinerea*. This combination of upregulated plant defense genes suggests that both SA and JA pathways were being induced in the transgenic plants (Reem et al., 2020).

Arabidopsis wild-type plants sprayed with *Fusarium graminearum* xylanase developed smaller lesions when infected with *P. syringae* compared to water mock treatment, but the lesions were the same after *B. cinerea* infection (Tundo et al., 2013). An inactive form of *Fusarium graminearum* xylanase was expressed transiently in tobacco, and these plants had smaller lesions from *P. syringae* but the same from *B. cinerea*. Arabidopsis plants transiently expressing *Fusarium* xylanase appeared normal compared to wild-type. The transgenic Arabidopsis developed smaller lesions compared to wild-type after treatment with *P. syringae* but not with *B. cinerea*. The xylanase did not inhibit *P. syringae* growth. Plant defense gene expression (*ORA59*, *PDF1.2*, *PR4*, and *PR1*) before and during *B. cinerea* infection was unaltered in the transgenic plants compared to wild-type. The same results were found when treated with *P. syringae*, except that *PR1* gene expression was higher 72 hours after inoculation. Additionally, the defense gene response and MAPK activation after OG and flg22 elicitation in the transgenic plants were also unchanged compared to the control. Wheat sprayed with xylanase before *F. graminearum* infection was more resistant early during infection and had less pathogen growth overall. In addition, wheat plants treated with xylanase accumulated more callose in comparison with wild-type but only after infection, and not before. Callose deposition early in the defense response could have a significant impact on pathogen growth and disease symptoms. Plants have different defense mechanisms against different types of pathogens. For example, the expression of *PR1* is not induced during *B. cinerea* infection. On the other hand, the defense genes *ORA59* and *PDF1.2* are upregulated during necrotrophic fungal infections, and these genes were similarly expressed in both wild-type and xylanase-expressing Arabidopsis plants. It was not surprising moreover that both the transgenic and wild-type plants were equally susceptible to the necrotroph *B. cinerea*. However, xylanase expressing plants and xylanase treatments appear to be effective in producing more resistant Arabidopsis, tobacco, and wheat against the hemibiotrophic fungi *P. syringae* and *F. Graminearum* (Tundo et al., 2013).

### 4.2 Transgenic plants expressing plant CW-modifying enzymes

Transgenic *Fragaria vesca* (wild strawberry) expressing the pectin methyl esterase *FaPE1* from *Fragaria ananassa* did not have any alterations in their phenotype (Osorio et al., 2008). Total PME activity increased in these transgenic plants, and the pectin was about 20% less methyl esterified compared to wild-type. The fruits of these transgenic lines were more resistant to *B. cinerea* infection, and the *PR5* defense gene expression was constitutively expressed in the transgenic fruits compared to the control. Additionally, 35% more salicylic acid (SA) was observed in the fruits compared to wild-type plants. Oligogalacturonides (OGs) isolated from the transgenic plants increased the expression of *PR5* and the accumulation of SA more than OGs isolated from wild-type. Completely de-esterified OGs did not elicit *PR5* gene transcription when inoculated on strawberry plants (Osorio et al., 2008). Additionally, OG from wild-type and transgenic strawberry did not elicit an immune response in *Nicotiana benthamiana*. It seems like there is a strawberry-specific receptor that allows the perception of the specific OGs. The degree of pectin methylation determines strawberry response to OGs (Osorio et al., 2008).

*Fragaria vesca* (wild strawberry) PME gene (*FaPE1*) was transformed into *Fragaria ananassa* (garden strawberry), and transformed plants had partially demethylated oligosaccharides (OGs) compared to wild-type (Osorio et al., 2011). The OGs extracted from the *FaPE1* overexpression lines showed upregulated plant defense responses; however, when de-esterified, OG treatment did not induce *PR5* gene expression. The transgenic plants had more phenylalanine, tryptophan, tyrosine, and aspartate, but less alanine, proline, and spermidine. Additionally, flavonols, flavanols, and flavanones were more abundant in the transgenic plants. The genes of several distinct functions had altered expression in the transgenic line, including carbon metabolism and unknown receptor-like kinases that were named *LRR1* and *LRR2*. Other characterized enzymes in this family are involved in pathogen perception and plant defense response. Additionally, genes similar to the *WRKY* gene family were identified and proposed to have transcription factor activity. Finally, a beta-xylosidase was found to be upregulated, which may be involved in CW remodeling in response to the pathogen. Less auxin was found in the fruit receptacle in transgenic fruit, which correlated with ripening. Overall, it was concluded that FaPE1 is responsible for producing active OGs that are perceived by the plant, and auxin may modulate a defense and growth tradeoff (Osorio et al., 2011).

*Actinidia chinesis* (kiwi) Pectin Methyl Esterase Inhibitor (AcPMEI) was transformed into wheat with the goal of inhibiting wheat PME activity (Volpi et al., 2011). Transformed wheat did not have any morphological changes compared to wild-type. There was less long and short de-esterified pectin in the transgenic lines compared to wild-type, and they were less susceptible to *B. sorokiniana* and *F. graminearum*. An *in vitro* study showed that highly methyl esterified pectins are less susceptible to the polygalacturonases of both *B. sorokiniana* and *F. graminearum* (Volpi et al., 2011).

Transgenic wheat expressing the bean PvPGIP2, which recognizes lots of diverse fungal PGs, was able to inhibit fungal PGs and had reduced disease symptoms to *F. graminearum* and *B. sorokiniana* infection (Ferrari et al., 2012).

PMEIs are effective against PMEs in other plant species as well. *Nicotiana tabcum* plants transiently expressing *Triticum aestivum* xylanase inhibitor-I (TAXI-I) and Arabidopsis plants stably expressing TAXI-I are more resistant to *Botrytis cinerea* compared to wild-type plants, however they were not resistant against *P. syringae* pv. *maculicola* (Tundo et al., 2020). When the xylanase BcXyn11a was inoculated on the leaf surface, necrotic lesions were reduced in transgenic plants that had a higher TAXI-I gene expression. Mixing the xylanase and purified TAXI-I together also prevent necrotic lesion formation on leaves suggesting that the TAXI-I is inhibiting the xylanase activity (Tundo et al., 2020).

## 5 Evaluation of various approaches

There are a variety of different approaches that can be taken to understand how the CW mediates plant immunity (**Figure 2**). A forward and reverse genetics approach is the most common method used so far. This method has been helpful in showing that altering each individual component of the CW has a different downstream effect on plant responses to pathogens and how the mechanisms involved in this altered response may or may not be mediated via immune pathways and stress hormones such as salicylic acid (SA), jasmonic acid (JA), or ethylene. One of the strengths of this approach has been using forward genetics screens and suppressor screens to discover novel CW modifications that are involved in plant immunity that may not be found otherwise. Not all CW-modifying enzymes contribute equally to plant immunity, but the most important ones can be identified and characterized using this approach. However, some mutants were not helpful in correlating CW composition with disease resistance either because the changes were too subtle to measure or because of redundant genes which makes it difficult to observe this correlation.

The application of inhibitors of CW-synthesizing enzymes has helped to confirm the results of phenotyping and bioassays in some genetic experiments because they can mimic the effect of knocking out a gene. However, one of the disadvantages is the possibility of plants experiencing unintended side effects from the treatment, which may influence the conclusions of the experiments. In these experimental approaches, careful controls must be used to determine the true effect of the inhibitor on the plant and defense responses.

Additionally, exogenous hydrolase treatments have been used to test the digestibility of different types of CWs in plants that have natural resistance or have altered resistance to pathogens. One of the advantages of this approach is that the CWs are being modified by enzymes that are secreted by pathogens. They further elucidate characteristics of the CW and how they are broken down by enzymes that are secreted by pathogens during infection. Also, this approach has helped to characterize the DAMPs that are released by CWDEs during pathogenesis, how DAMPs are perceived by plants, and how they induce plant immune responses. There is much more to explore in terms of characterizing DAMPs that are produced by different CWDEs in different plant species, what responses DAMPs elicit, how plants perceive DAMPs, and what immune pathways are used. There are technical challenges of this approach including isolating pure cell wall components and functional CWDEs.

**Figure 3:**
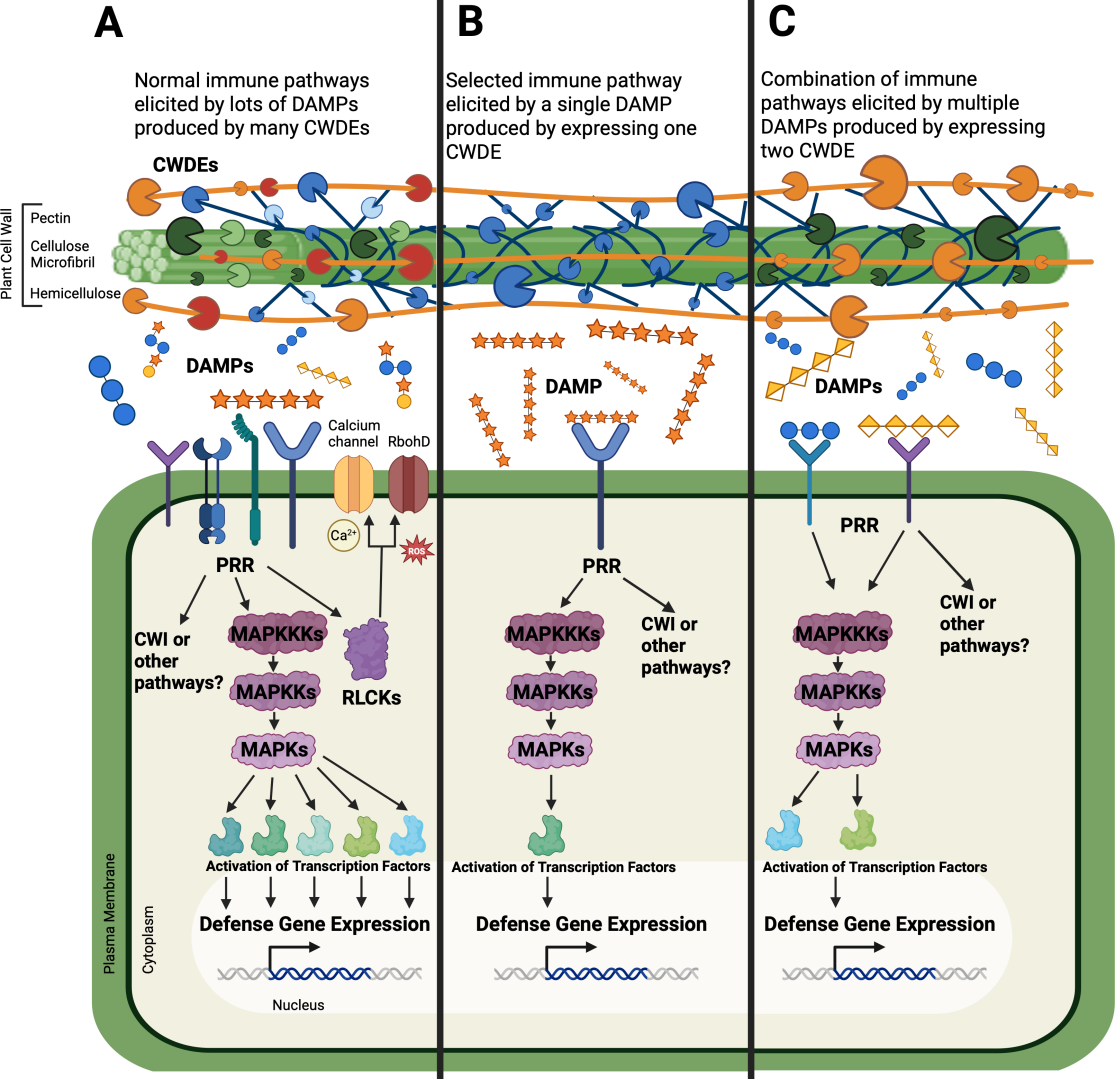
The transgenic plants expressing cell wall degrading enzymes (CWDEs) can allow for a more selective elicitation of the plant defense response. Normally during infection, pathogens secrete many different CWDEs that produce a host of damage-associated molecular patterns (DAMPs) from the CW, eliciting many immune pathways in the plant. DAMPs are perceived by pattern recognition receptors (PRRs) which can activate receptor-like cytoplasmic kinases (RLCK) to initiate calcium signaling (Ca^2+^) or reactive oxygen species (ROS) production through RbohD (respiratory burst oxidase homolog D). Additionally, mitogen-activated protein kinase (MAPK) can be phosphorylated to change defense gene expression, or the cell wall integrity (CWI) can be compromised inducing other changes. To isolate these responses, the expression of a single CWDE gene in plants can produce a single DAMP, eliciting only a few pathways. CWDE genes can also be expressed in specific combinations.

Finally, the heterologous expression of CW-modifying enzymes *in planta* is a promising approach (**Figure 3**). These enzymes are frequently sourced from both pathogens and plants. When pathogen-sourced genes are used, this method has the advantage of modifying the CW in the same way that would occur during pathogenesis, thus mimicking the action of microbial CWDEs (**Figure 3**). Additionally, many different CWDEs are secreted by pathogens, inducing lots of downstream immune pathways (**Figure 3A**). Specific changes in the cell wall can be analyzed one at a time by expressing individual CWDE genes, separating out individual immune pathways that are induced by the specific change (**Figure 3B**). Such post-synthetic CW modification, in contrast to mutants or overexpression lines of genes encoding synthetic enzymes, has a significant advantage, taking place in the apoplast and not inside the plant cytoplasm or the Golgi. This approach avoids negative impacts on the plant morphology frequently observed in knockout mutants. One of the avenues of this approach that has just started to be explored is how combinations of enzymes work together to alter the CW and the host immune response (**Figure 3C**). Additionally, it would be interesting to use an inducible promoter for these transgenes to explore what short-term immune responses occur when the expression is initiated at certain time points instead of the continuous presence of constitutively expressed enzymes.

Overall, understanding the exact molecular mechanisms of CW-mediated signals and how that translates to immune responses, such as defense gene expression, is an actively developing area of research. There are several plasma-membrane pattern recognition receptors that are proposed to bind to DAMPs derived from the CW (Molina et al., 2024). However, there are potentially more PRRs that have not yet been identified (Molina et al., 2024). Recent discoveries have shown interesting connections between CW integrity (CWI) and pattern-triggered immunity (PTI, Engelsdorf et al., 2018; Gigli-Bisceglia et al., 2020; Bellincampi et al., 2014). How these signals are integrated depends on both the plant and pathogen species (Bellincampi et al., 2014). The DAMP PEP1 inhibits CWI signaling (Engelsdorf et al., 2018). Additionally, when PTI signaling is impaired, CWI signaling increases to compensate for the loss (Engelsdorf et al., 2018). FER may coordinate these signaling pathways receiving multiple inputs from the plasma membrane and regulating multiple downstream responses (Gigli-Bisceglia et al., 2020). Alteration in cellulose biosynthesis can induce both osmo-perception and mechano-perception which induce defense signaling downstream (Gigli-Bisceglia et al., 2020). It is now largely understood and accepted that the CW is dynamic and involved in the perception and response of both biotic and abiotic stressors, but understanding the exact dynamics of the CW, how it is regulated, and what downstream cellular responses CW alterations cause is still a frontier that needs to be explored, requiring broader and diverse approaches.
